# Identification of an unusual *Brucella *strain (BO2) from a lung biopsy in a 52 year-old patient with chronic destructive pneumonia

**DOI:** 10.1186/1471-2180-10-23

**Published:** 2010-01-27

**Authors:** Rebekah V Tiller, Jay E Gee, David R Lonsway, Sonali Gribble, Scott C Bell, Amy V Jennison, John Bates, Chris Coulter, Alex R Hoffmaster, Barun K De

**Affiliations:** 1 Division of Foodborne, Bacterial, and Mycotic Diseases and Division of Healthcare Quality Promotion, Centers for Disease Control and Prevention, 1600 Clifton Road, Atlanta, GA 30333, USA; 2 Infection Management Service and Microbiology, The Prince Charles Hospital, Rode Road Chermside, Queensland 4032, Australia; 3 Pathology Queensland Central Laboratory, Royal Brisbane and Women's Hospital, Herston Road, Queensland 4029, Australia; 4 Clinical and Statewide Services Division, Queensland Health, 39 Kessels Road, Coopers Plains, Queensland 4108, Australia

## Abstract

**Background:**

Brucellosis is primarily a zoonotic disease caused by *Brucella *species. There are currently ten *Brucella *spp. including the recently identified novel *B. inopinata *sp. isolated from a wound associated with a breast implant infection. In this study we report on the identification of an unusual *Brucella*-like strain (BO2) isolated from a lung biopsy in a 52-year-old patient in Australia with a clinical history of chronic destructive pneumonia.

**Results:**

Standard biochemical profiles confirmed that the unusual strain was a member of the *Brucella *genus and the full-length 16S rRNA gene sequence was 100% identical to the recently identified *B. inopinata *sp. nov. (type strain BO1^T^). Additional sequence analysis of the *recA, omp2a *and *2b *genes; and multiple locus sequence analysis (MLSA) demonstrated that strain BO2 exhibited significant similarity to the *B. inopinata *sp. compared to any of the other *Brucella *or *Ochrobactrum *species. Genotyping based on multiple-locus variable-number tandem repeat analysis (MLVA) established that the BO2 and BO1^T^strains form a distinct phylogenetic cluster separate from the other *Brucella *spp.

**Conclusion:**

Based on these molecular and microbiological characterizations, we propose that the BO2 strain is a novel lineage of the newly described *B. inopinata *species.

## Background

Brucellosis is primarily a zoonotic disease, caused by members of the genus *Brucella*, which currently constitutes several species based on pathogenicity, host preferences and phenotypic characteristics: *B. abortus *(cattle), *B. canis *(dogs), *B. melitensis *(goats), *B. suis *(pigs), *B. ovis *(rams), *B. neotomae *(desert rats), *B. ceti *and *B. pinnipedialis *(marine mammals), and *B. microti *(common vole) [1-6]. Recently, a novel species, *Brucella inopinata*, associated with a human infection has been recognized as the newest member of the genus *Brucella *[7,8]. In early 1985, whole genome hybridization analysis studies revealed a high degree of genetic homology among the *Brucella *species, which led to the proposal that the genus *Brucella *was a mono-specific species with *B. melitensis *being the primary species and all others as sub-species and biovars [9-11]. However, due to the limited acceptability of the one-species concept, the traditional classification of *Brucella *spp. based on phenotypic characteristics has been re-instated by the *Brucella *Taxonomy Subcommittee in 2006 [3].

*Brucella *are facultative intracellular pathogens that infect many organs and soft tissues, including mammary glands. Infection frequently results in abortion, low milk production and fetal death in animals [2,12-16]. Brucellosis in humans is mostly caused by *B. abortus, B. melitensis, B. suis*, and sometimes *B. canis *[14,17-19], and is commonly associated with the consumption of unpasteurized dairy products, meat from infected animals and exposure to infected animal tissues or laboratory transmission [1,2,20]. Human brucellosis is a chronic debilitating infection with a very broad clinical picture potentially affecting any major organ, including the lung, causing varying respiratory symptoms [20]. Respiratory infections in humans caused by *Brucella *spp. is a rare manifestation with reports describing multifocal abscesses or nodules, hilar adenopathy and hemorrhagic pleural effusion with resolution by antimicrobial therapy and lung decortications [21-26]. Most pulmonary brucellosis cases were found in farmers handling infected meat or travelers who consumed raw infected animal meat or unpasteurized milk products while visiting countries endemic for brucellosis [26,27].

We report the isolation and identification of an unusual gram-negative, non-motile *Brucella*-like coccoid bacillus (BO2) isolated from a lung biopsy in a 52-year-old male in Australia with a history of chronic destructive pneumonia. The patient traveled worldwide but denied any common risk factors associated with brucellosis. Both biochemical and molecular characteristics of the BO2 strain have demonstrated unique similarity with a recently described *B. inopinata *strain (BO1^T^) associated with a breast implant wound of a 71-year-old patient from Portland, Oregon with clinical signs of brucellosis [7,8].

## Results

### Phenotypic characterization

BO2 cells grown on SBA or RBA at 35-37°C with or without 5% CO_2 _for 24 to 48 h were circular, convex, entire, smooth and opaque. The organisms were gram-negative, generally stained uniformly; and appeared coccoid to short coryneform rods. Colonies of the BO2 strain ranged in size from punctuate to 1.5 mm in diameter and they were non-motile, mucoid colonies on MacConkey agar; positive for oxidase and catalase, exhibited nitrate reduction with production of gas and rapid urease production (< 5 min). Hydrogen sulfide production by the BO2 strain was observed by the development of a dark gray color on lead acetate paper suspended above the heart infusion agar slant. Subculture of individual colony types produced similar profiles and no hemolytic reaction was observed on SBA plates after overnight incubation at 37°C. The BO2 cells grew in the presence of thionine (1:25,000, 1:50,000 and 1:100,000 dilutions) and basic fuchsin (1:50,000 and 1:100,000 dilutions) dyes within 24 to 48 h. Both the acriflavin and gel formation tests were negative. However, lysis by Tbilisi phage specific for detection of *Brucella *spp. in two routine test dilutions (1× and 4× RTD) appeared incomplete [7,8,28] and agglutination of the BO2 cells with either monospecific anti-M or anti-A antisera were very weak.

### Antimicrobial susceptibility test

The antimicrobial susceptibility profile of the BO2 strain was compared with a set of 93 other *Brucella *spp. strains (74 *B. melitensis*, 14 *B. suis *and 5 *B. abortus*) along with BO1^T ^based on CLSI interpretive requirements for *Brucella *spp. [8,29,30]. Both strains had very similar MIC patterns to all *Brucella *reference strains tested previously [8,30] (Table 1). BO1^T ^and BO2 strains grew well in cation-adjusted Mueller-Hinton broth (CAMHB) after just 20 hours of incubation, unlike other *Brucella *spp. (e.g., *B. abortus*, *B. melitensis*, and *B. suis*) which do not routinely grow very well in CAMHB and require 48 hours of incubation in *Brucella *broth for MIC testing [30]. Our standard phenotypic characterization, including the antimicrobial susceptibility profiles, suggested that the BO2 strain more closely resembled the BO1^T ^strain of the *B. inopinata *sp. than the other classical *Brucella *spp.

**Table 1:** MIC results for 5 antimicrobial agents tested against BO1^T^, BO2 strains and 93 *Brucella *strains

	BO1^T ^MIC (μg/ml)			BO2 MIC (μg/ml)			Brucella spp.^a ^in *Brucella *broth 48 h	
	CAMHB^b^	*Brucella *Broth	*Brucella *Broth	CAMHB	*Brucella *Broth	*Brucella *Broth	MIC Range	MIC_90_
**Antimicrobial agent**	20 h	20 h	48 h	20 h	20 h	48 h	(μg/ml)	(μg/ml)
Doxycycline	0.25	0.25	0.5	0.25	0.25	0.5	0.06 - 1	0.25
Gentamicin	1	2	2	1	2	2	0.5 - 2	1
Streptomycin	4	4	4	2	4	4	1 - 8	4
Tetracycline	0.25	0.5	1	0.12	0.25	0.25	0.12 - 1	0.5
Trimethoprim-sulfamethoxazole	0.5/9.5	0.25/4.75	0.5/9.50.25	0.5/9.5	0.25/4.75	0.5/9.5	0.12/2.38 - 0.5/9.5	0.5/9.5

^a ^Ninety-three *Brucella *isolates (74 *B. meli1tensis*, 14 *B. suis*, and 5 *B. abortus*) were tested [30].

^b ^CAMHB = cation-adjusted Mueller-Hinton broth.

### Molecular characterization

#### Detection of IS*711 *element by PCR

**Figure 1:**
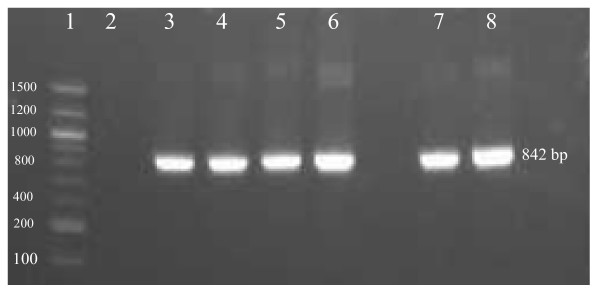
**IS*711 *profiles of PCR amplified products analyzed by gel electrophoresis on a 2% E-Gel displaying the following: molecular weight marker (lane 1), no template control (lane 2), *B. abortus *ATCC 23448 (lane 3), *B. melitensis *16 M (lane 4), *B. suis *ATCC 23444 (lane 5), *B. ovis *ATCC 25840 (lane 6), BO1^T ^(lane 7), and BO2 (lane 8)**.

The *Brucella *specific insertion sequence (IS*711*) PCR was performed amplifying an 842-bp repetitive element using BO2 genomic DNA. The IS*711 *profile observed in strain BO2 was approximately the same size as that of the BO1^T ^strain and the classical *Brucella *spp. including *B. ovis *(ATCC 25840) (Figure 1). The BO2 strain also generated several large amplicons (>1000 bp) similar to BO1^T ^and other *Brucella *strains with low intensity as reported earlier [8].

#### Real-Time PCR for BO1^T^/BO2

A TaqMan PCR assay targeting conserved regions of the BO1^T ^and *Brucella *spp.16S rRNA gene sequence was designed for rapid differentiation of potential *B. inopinata-*like strains from all other classical *Brucella *and *Ochrobactrum *spp. This real-time PCR assay, using two hybridization probes: BI-P specific for *B. inopinata *spp. and BRU-P specific for *Brucella/Ochrobactrum *spp., gave average crossing threshold (Ct) values in the range of 15 to 20 (strong positive). The BI-P probe demonstrated perfect agreement for both BO1^T ^and BO2 strains as did the BRU-P probe for all other *Brucella *or *Ochrobactrum *spp. respectively. Both probes showed no cross reactivity against the other non-*Brucella *strains tested to date [31] demonstrating very high specificity of the target sequences in the PCR assay. Both the BO1^T^/BO2 and the *Brucella/Ochrobactrum *specific probes were capable of optimal detection of template down to 10 fg/μl concentration of genomic DNA template (data not shown).

#### 16S rRNA gene sequence analysis

Rapid identification of the BO2 strain as *B. inopinata-*like by the BO1 PCR assay led to sequence analysis of the full-length 16S rRNA gene (1,412 bp) of the BO2 strain. Full sequence alignment with the 16S rRNA gene sequences of BO1^T^, reference *Orchrobactrum *spp. strains, and the *Brucella *spp. consensus sequence confirmed that the BO2 strain shared 100% 16S rRNA gene sequence identity to that of BO1^T ^and 99.6% identity with other *Brucella *spp. (Table 2).

**Table 2:** Comparative percent identity based on pair-wise analysis of five genes of BO2 with BO1^T ^and classical *Brucella *spp. using MEGA4.

BO2 genes	*B. inopinata *BO1^T ^(%)	*Brucella *spp. (%)
16S rRNA	100.0	99.6
*RecA*	98.2	99.2
MLSA	98.7	98.3-98.6
*Omp2a*	99.0	85.4-98.4
*Omp2b*	95.3	83.8-95.3

#### *Omp2a/2b *genes sequence analysis

**Figure 2:**
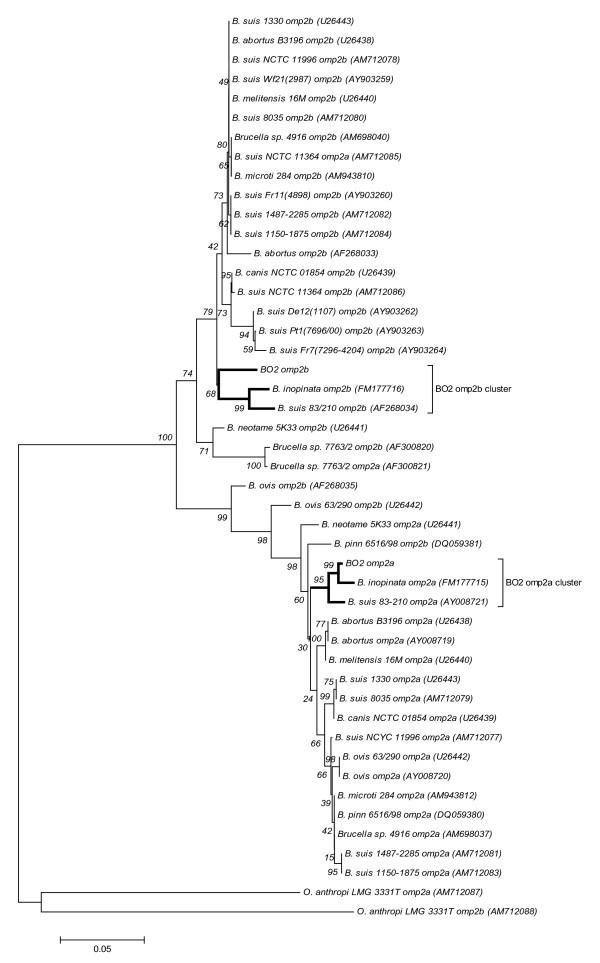
**Phylogenetic tree reconstructed with *omp2a *(1093 bp) and *omp2b *(~1211 bp) sequences using MEGA v.4.0 neighbor joining analysis**. The bootstrap consensus tree inferred from 1000 replicates is taken to represent the evolutionary history of the taxa analyzed. The significance of each branch is indicated by a bootstrap percentage calculated from 1000 replicates.

We also analyzed two highly homologous outer membrane porin genes (*omp2a *and *omp2b*) of the BO2 and BO1^T^strains and compared their full-length sequences with that of other *Brucella *species available in GenBank. The phylogenetic relationships derived by neighbor-joining clustering analysis of the BO2 *omp2a *(1093 bp) and *omp2b *(~1212 bp) genes with the NCBI sequences of other *Brucella *strains and the *Ochrobactrum anthropi *LMG 3331 reference strain demonstrated considerable intra- and inter-species variability (Figure 2). The BO2 *omp2a *and *omp2b *genes are 84.6% homologous to each other. Neighbor-joining clustering analysis of both *omp2a *and *omp2b *nucleotide sequences shows that BO2 clusters closest to BO1^T ^and an atypical *B. suis *83-210 strain [32]. The *omp2a *gene of BO2 is only 1.0% divergent from that of BO1^T^. The *omp2b *gene is characteristically more diverse within the *Brucella *spp. and is also evident with the BO2 *omp2b *gene which was 95.3% and 94.1% identical to the BO1^T ^and *B. suis *83-210 strains, respectively (Figure 2, Table 2). Clustering analysis demonstrates that BO1^T^, BO2 and the *B. suis *83-210 strains form consistent sub-groups based on their *omp2a *and *omp2b *gene homology [32].

#### *RecA *gene sequence analysis

**Figure 3:**
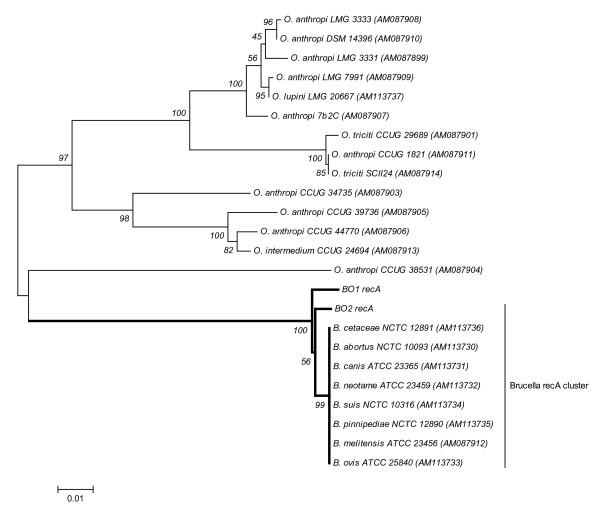
**Phylogenetic tree reconstructed with *recA *(948 bp) sequences using MEGA v.4.0 neighbor joining analysis**. The bootstrap consensus tree inferred from 1000 replicates is taken to represent the evolutionary history of the taxa analyzed. The significance of each branch is indicated by a bootstrap percentage calculated from 1000 replicates.

The *recA *gene (948 bp) of strain BO2 was compared to those of BO1^T^, the classical *Brucella *spp.(n = 8) and several representative *Ochrobactrum *spp. [31,33]. Within the genus *Brucella*, the *recA *gene is highly conserved with 100% nucleotide sequence identity among the different species. Interestingly, the BO2 *recA *nucleotide sequence reveals 99.2% identity to the *Brucella *consensus *recA *sequence due to 8 nucleotide substitutions. However, the BO2 *recA *gene has a lower identity (98.2%) when compared to the BO1^T ^*recA *sequence differing by 17 nucleotides. Phylogenetic analysis of BO1^T ^and BO2 strains with other *Brucella *and *Ochrobactrum *spp. shows that the *Brucella *spp. clade including BO2 and BO1^T^, are distantly similar to the *Ochrobactrum *spp. with approximately 85% sequence identity (Figure 3).

#### Multiple Locus Sequence Analysis

**Figure 4:**
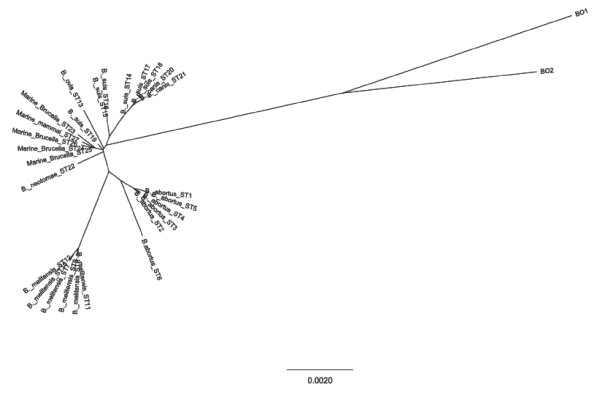
**Unrooted phylogenetic reconstruction of the concatenated sequences of nine house-keeping genes (4,396 bp) using the neighbor-joining approach**. Represented are the 27 known *Brucella *sequence types along with BO1^T ^and their relation to BO2.

Multiple locus sequence analysis (MLSA) of nine *Brucella *spp. house-keeping genes has been used to differentiate *Brucella *spp. into distinct sequence types (ST). BO1^T ^was determined to be 1.67% divergent from ST1 and to possess novel alleles at all nine loci [8]. BO2 has shown similar divergence (1.5%) from ST1 by MLSA also with novel alleles in all nine loci. Neighbor-joining phylogenetic analysis clearly shows how divergent the BO1^T ^and BO2 species are from the classical *Brucella *sequence types (Figure 4). Throughout the 4,396-bp sequence examined, the BO1^T ^and BO2 genomes have 32 common SNPs while there are 30 BO1^T ^and 26 BO2 specific nucleotide changes that further characterize the divergence of these two strains at these highly conserved loci in the *Brucella *genus.

#### Multiple-Locus Variable-Number Tandem Repeat Analyses

**Figure 5:**
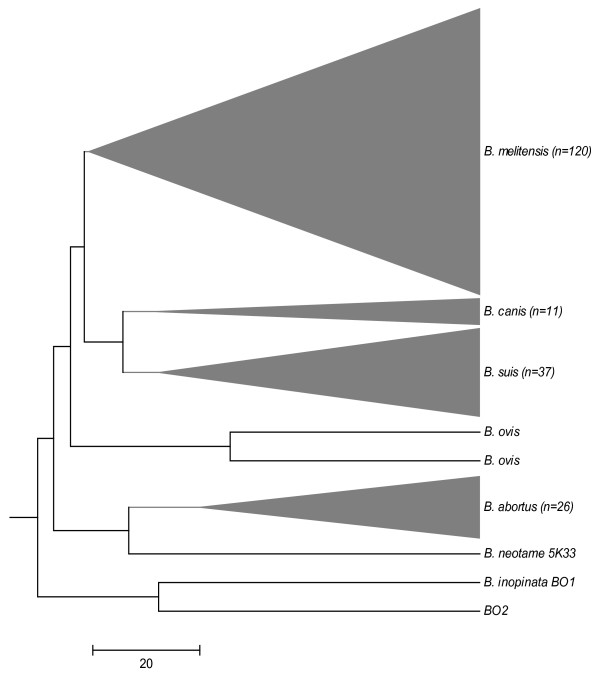
**Condensed unweighted pair group method analysis (UPGMA) dendogram of multiple-locus variable number tandem repeat analysis (MLVA) genotypes of BO1^T^, BO2 strains along with 209 characterized *Brucella *strains**.

Both BO2 and BO1^T ^strains were also investigated by multiple-locus variable-number tandem repeat (VNTR) analysis (MLVA) using fifteen VNTR loci by capillary electrophoresis. Results were compared with a panel of well-characterized *Brucella *strains (n = 209) representing known species from our collection [31]. Our MLVA-15 typing analysis of both BO2 and BO1^T ^strains demonstrated unique VNTR profiles in which both strains have six *Brucella*-loci with the same alleles (VNTR 2, -3, -14, -20, -21 and -25); and seven loci with variable VNTR amplicons (VNTR1, -7, -27, -29, -30, -31 and -33). All VNTRs successfully amplified in both BO1 and BO2 with the exception of VNTR16 and -28 in BO1^T^. MLVA-15 analysis revealed that both BO2 and BO1^T ^had distinct VNTR profiles in comparison to each other and other *Brucella *strains (Figure 5).

## Discussion

In this paper we present the identification of an atypical *Brucella*-like strain (BO2) isolated from the lung biopsy of a 52-year-old patient. As a young adult he lived in Oregon on two occasions (1981 and 1985-1987), and experienced an unexplained 'liver failure' and then severe pneumonia (with pleurisy) from which he recovered with multiple courses of antimicrobial therapy as reported by the patient to his physicians in Australia. This patient was originally misdiagnosed because of the misidentification of the BO2 strain as *O. anthropi *on an AP1 20NE system. It is a common practice for clinical labs to attempt rapid identification of gram-negative *coccobacillus *organisms like *Brucella *spp. from blood culture using automated systems. However, the *Brucella *spp. are often misidentified due to their similar phenotypic characteristics to closely related organisms such as *Ochrobactrum *spp. [34,35]. Though the patient was initially treated for both *Ochrobactrum *and *Brucella *infections due to the difficulties in diagnosis, he recovered with an extended course of combination oral antimicrobial therapy.

This BO2 strain is phenotypically and molecularly similar to the recently identified *B. inopinata *type strain (BO1^T^) recovered from a patient from Oregon, which was also originally misidentified as *O. anthropi *by the API 20E and API 20NE [7,8]. Both these strains share common colony morphology and biochemical characteristics including rapid urease and positive H_2_S production, inability or very weak agglutination with *Brucella *specific antisera for the lipopolysaccharide-O-antigens or acriflavin. Neither the BO1^T ^or BO2 strains supports gel formation or exhibits growth inhibition to the dye media as shown by common members of the genus *Brucella*. BO2 also exhibited incomplete lysis by Tbilisi phage and had very similar antimicrobial susceptibility profiles to BO1^T ^in comparison to other *Brucella *reference strains.

Insertion sequence (IS) fingerprinting in the *Brucella *species has shown that the genomic localization and copy number of the IS*711 *insertion element (also called IS*6501*) is species-specific and could have an association with specific pathogenicity for a preferred host [36-38]. The presence of multiple copies of BO1^T^-like IS*711 *insertion sequences suggest not only that BO2 is a member of the *Brucella *genus (Figure 1) but that the BO2-IS*711 *amplification pattern specifically resembles that of the newly described *B. inopinata *species [8]. Positive identification of the BO2 strain as a member of *B. inopinata *by our real-time BO1 PCR assay was significant. Both BO1^T ^and BO2 strains were the cause of distinct and unusual forms of human brucellosis. Atypical clinical isolates of this nature can often be misdiagnosed by automated systems as was the case with BO1^T ^and the BO2 strain described here [8,35]. The availability of the real-time TaqMan assay served as a reliable first-line tool for determining *B. inopinata-*like species.

These initial findings led to further characterization and sequence-based typing which provided additional supporting evidence that this new BO2 strain most resembles the *B. inopinata *sp. within the *Brucella *genus. Using broad-range eubacterial primers, Gee *et. al*. effectively demonstrated the advantage of 16S rRNA gene sequencing to identify *Brucella *isolates reporting 100% identity in all the strains examined [31]. Interestingly, the full-length 16S rRNA gene sequence of BO2 was 100% identical to that of BO1^T ^and 99.6% identical to the *Brucella *spp. consensus 16S rRNA gene sequence. The high sequence identity of the BO2 16S rRNA sequence to the recently described *B. inopinata *sp. is remarkable and represents the first recognized *Brucella *species to have a divergent 16S rRNA sequence [8].

The *recA *gene has been investigated as an alternative phylogenetic marker for several bacterial genera due to its highly conserved nature and ubiquity in prokaryotes [33,39,40]. Unlike the high sequence homology of the *recA *gene within the *Brucella *genus [41], we identified unique variability in the *recA *gene sequences of BO2 and BO1^T^. Sequence analysis revealed that the *recA *nucleotide sequence of the BO2 strain shared greater similarity with the *Brucella *spp. *recA *consensus sequence than to BO1^T^. Both BO2 and BO1^T ^*recA *sequences are distanced by 8 and 11 unique SNPs, respectively, from the *Brucella *spp. *recA *consensus sequence, and share only one common transversion at the 517 nucleotide position. Translation of the *recA *gene sequences of BO1^T^, BO2 and the *Brucella *spp. consensus sequence shows that all base pair changes were synonymous substitutions having no effect on protein structure or function. The *Brucella *outer membrane proteins have been studied extensively for their function in virulence, pathogenicity, bacteriophage reception, antigenic factors and antibacterial evasion [42-45]. The genetic variability among the *omp *genes within the *Brucella *spp. has proven effective at characterizing *Brucella *spp. and strain types and is often used for higher resolution molecular typing [4,32,43,45]. The *omp2a/2b *genetic analysis we report here is very interesting in that BO2 consistently associates with not only BO1^T ^but the atypical *B. suis *83-210 strain that was isolated from a rodent in Australia [32]; and thus further investigation may be warranted into rodents as a possible natural reservoir for these novel *Brucella *species.

Investigation of the nine housekeeping genes by multi locus sequencing analysis demonstrates that BO2 is genetically distinct from BO1^T ^yet exhibits remarkably similar divergence (1.5%) from the classical *Brucella *sequence types as shown in Figure 4. The relative similarity of the nucleotide sequences of BO1^T ^and BO2 by MLSA demonstrates uniquely distant sequence types within the currently characterized *Brucella *spp. and should be considered as a new group of STs within the *Brucella *genus. They also exhibit distinct allelic profiles by MLVA although all alleles in both the BO1^T ^and BO2 allelic profiles have been observed in other *Brucella *spp. Furthermore, the phylogenetic analysis shown in Figure 5 demonstrates that these strains form a single separate cluster from the classical *Brucella *spp. [8].

The molecular and microbiological characteristics presented here provide supporting evidence that strain BO2 is most closely associated with the BO1^T ^strain and should be considered as a novel lineage of *B. inopinata *sp. Attempting to understand the evolutionary origin of these two strains is somewhat confounded by the interesting and disparate medical histories of the case patients (who both happened to have lived in Portland, Oregon) from whom these strains were isolated and suggests that there are new and emerging *Brucella *strains capable of causing unusual presentation of human brucellosis.

## Conclusion

Phenotypic and genomic analysis of the unusual *Brucella *strain (BO2) from a lung biopsy have established it as a lineage of the recently identified novel *B. inopinata *sp. type strain BO1^T^, which was isolated from a wound associated with a breast implant. This is the first report of a human brucellosis case associated with chronic destructive pneumonia caused by an atypical *Brucella *strain. An interesting finding from our molecular analysis reveals that both strains BO1^T ^and BO2 appeared to be closely related to a less-characterized *B. suis *strain 83-210 (isolated from a rodent in Australia) by their *omp2a/2b *genes, which may suggest a common ancestor and may also provide insight into the ecological niche, and host reservoir for these novel *Brucella *strains causing unusual human infections.

## Methods

### Patient

The patient was born in Malta in 1956 and immigrated to Australia at age two, where he would continually return and eventually settle throughout extensive worldwide travel including the Western region of the United States. Between 2003 and 2007, the patient was hospitalized multiple times in different hospitals in Australia for abnormal liver function, community acquired pneumonia, anterior chest wall abscess and sinus infection. In September 2007 a percutaneous lung biopsy was performed and a gram-negative organism was isolated from a broth culture of the fine needle aspirate of the patient's lung and identified as *Ochrobactrum anthropi *on an API20NE system. The testing laboratory was aware of the possibility of *Brucella *sp. being misidentified as *Ochrobactrum anthropi *[35] and the isolate was referred for further testing. The patient was treated with combination therapy of doxycycline and rifampicin for twelve months and ciprofloxacin for three months (the latter was ceased after molecular testing confirmed *Brucella *species). The culture was initially tested according to standard microbiological and molecular procedures and then forwarded to the Centers for Disease Control and Prevention (CDC), Atlanta, GA, for further characterization. This gram-negative organism was designated as BO2 and stored at -70°C in defibrinated rabbit blood until further evaluation.

### Phenotypic analysis

The BO2 strain was routinely maintained on Trypticase soy agar with 5% defribinated sheep blood agar (SBA) or rabbit blood agar (RBA) (BBL Microbiology Systems, Cockeysville, MD). Phenotypic identification of the BO2 strain was performed according to the laboratory techniques in brucellosis described by Alton *et. al*. in the World Health Organization monogram [7,8,28].

### Antimicrobial susceptibility analysis

The antimicrobial susceptibility testing of the BO2 strain was performed by the broth microdilution method in CAMHB and *Brucella *broth in accordance with the Clinical and Laboratory Standards Institute (CLSI) protocol as described previously [8,29]

### Molecular analysis

#### Detection of IS*711*

To detect the *Brucella-*specific insertion sequence IS*711 *element (842 bp) [37], cell lysate DNA templates from strains BO2, BO1^T^, *B. abortus *(ATCC 23448), *B. suis *1330 (ATCC 23444), *B. ovis *(ATCC 25840) and *B. melitensis *16 M (ATCC 23456) were amplified and the amplicons were analyzed by 2% E-Gel agarose gel electrophoresis as mentioned previously [8].

#### Real-Time PCR assay

A real-time TaqMan PCR assay was developed targeting a four base nucleotide substitution within the 16S rRNA gene sequence of BO1^T ^(positions 145 to 148; GenBank accession no. EU053207). We designed two PCR primers16SF (5'-CGGGCCGATCATTTGC-3') and16SR (5'-AACTCAGGGAAACTTGTGCTAATACC-3') to amplify a 72-bp region of the 16S rRNA *Brucella *consensus sequence and two hybridization probes, BI-P (5'-AAATCTTTCCCCTTTCGGGCAC-3') and BRU-P (5'-AAATCTTTCCCCCGAAGGGCAC-3'), targeting a 4-bp polymorphic region within the 72-bp amplicon. Both probes were synthesized with a 6-carboxyfluorescein reporter molecule attached at the 5' end and Black Hole Quencher 1 on the 3' end. Each final PCR reaction mix contained 2 μl of DNA template and 18 μl of PCR master mixture containing 1 × LightCycler Faststart DNA Master HybProbe mix (Roche Applied Sciences, Indianapolis, IN), 4 mM MgCl_2_, 0.4 μM of each primer and 0.2 μM of probe. The LightCycler thermal cycling conditions were 95°C for 8 min followed by 45 cycles of 95°C for 5 sec and 60°C for 5 sec ending in a 45°C hold for 1 min 15 sec. A panel of 54 well characterized *Brucella *strains and 28 near-neighbors, including 5 *Ochrobactrum *strains [31] were evaluated by the assay. Positive results are expressed in log scale as crossing threshold values (Ct) of fluorescence released above the no-template control baseline of 0.01 following each amplification as described by the manufacturer.

#### 16S rRNA gene analysis

The full length amplicon of 16S rRNA gene was generated using the BO2 cell-lysate DNA and sequenced using the BigDye terminator cycle 3.1 sequencing kit (ABI, Foster City, CA) as described previously [31]. A comparative full-length sequence analysis of BO2 was performed with the consensus 16S rRNA gene sequence of *Brucella *spp. [31], and the *Ochrobactrum intermedium *type strain (GeneBank accession no. AM114411T) along with that of the *B. inopinata *BO1^T ^strain (GeneBank accession no. EU053207) using the GCG Wisconsin software package (version 10.2; Accelrys, San Diego, CA) and MEGA 4.0 [31,46].

#### *Omp2a/2b *and *recA *genes analysis

The full-length outer membrane porin genes *omp2a *and *omp2b*, and also the *recA *gene of BO2 were sequenced [33,45], and compared with sequences of BO1^T ^and other *Brucella *and *Ochrobactrum *spp. available in GenBank. Contigs were assembled and edited before multiple sequence alignments were constructed in the DNASTAR Lasergene 8 genetic analysis software suite (DNASTAR Inc., Madison, WI). Neighbor-joining consensus trees inferred from 1000 bootstrap replicates were constructed using MEGA version 4.0 [46].

#### MLSA typing

To assess the relation of BO2 with other classical *Brucella *spp. and BO1^T^, the multi locus sequence analysis (MLSA) primer sets were used to amplify and sequence nine discrete house-keeping genes as described previously [47]. Multiple sequences were aligned and neighbor-joining phylogenetic trees were constructed as described above.

#### Sequence identities

Similarity values reported throughout the text and in Table 2 were calculated from estimates of evolutionary divergence between the sequences represented in the corresponding dendograms. All results are based on the pairwise analysis of inclusive sequences using the Maximum Composite Likelihood method in MEGA 4.0 [46]. All positions containing gaps and missing data were eliminated from the dataset.

#### MLVA typing

Molecular typing of the BO2 strain based on multiple-locus variable-number tandem repeat (VNTR) analysis (MLVA) was investigated by examining fifteen *Brucella *spp. VNTR genetic markers (MLVA-15) [48,49], and a distance tree was generated in BioNumerics v.5.1 (Applied Maths, Saint-Martens-Latem, Belgium) by clustering analysis using the unweighted-pair group method with arithmetic averages (UPGMA) and saved in newick format. Tree manipulations and labeling were done in MEGA 4.0 [46].

## Authors' contributions

SG, SCB, AJ, JB CC participated in the clinical diagnosis, isolation and initial characterization of the strain BO2 and also contributed in drafting the manuscript. RVT, JEG, DRL, ARH, BKD performed both biochemical and molecular studies and drafted the manuscript. All authors read and approved the final manuscript.

## References

[B1] Boschiroli ML Foulongne V O'Callaghan D Brucellosis: a worldwide zoonosis Curr Opin Microbiol 2001 4 1 58 64 10.1016/S1369-5274(00)00165-X 11173035

[B2] Corbel MJ Brucellosis: an overview Emerg Infect Dis 1997 3 2 213 221 10.3201/eid0302.970219 9204307 PMC2627605

[B3] Osterman B Moriyon I International Committee on Systematics of Prokaryotes; Subcommittee on the taxonomy of *Brucella: *Minutes of the meeting, 17 September 2003, Pamplona, Spain Int J Syst Evol Microbiol 2006 56 1175 10.1099/ijs.0.64349-0

[B4] Cloeckaert A Verger JM Grayon M Paquet JY Garin-Bastuji B Foster G Godfroid J Classification of *Brucella *spp. isolated from marine mammals by DNA polymorphism at the omp2 locus Microbes Infect 2001 3 9 729 738 10.1016/S1286-4579(01)01427-7 11489421

[B5] Jahans KL Foster G Broughton ES The characterisation of *Brucella *strains isolated from marine mammals Vet Microbiol 1997 57 4 373 382 10.1016/S0378-1135(97)00118-1 9444074

[B6] Scholz HC Hubalek Z Sedlacek I Vergnaud G Tomaso H Al Dahouk S Melzer F Kampfer P Neubauer H Cloeckaert A *Brucella microti *sp. nov., isolated from the common vole Microtus arvalis Int J Syst Evol Microbiol 2008 58 Pt 2 375 382 10.1099/ijs.0.65356-0 18218934

[B7] Scholz HC Nockler K Gollner C Bahn P Vergnaud G Tomaso H Al Dahouk S Kampfer P Cloeckaert A Maquart M *Brucella inopinata *sp. nov., isolated from a breast implant infection Int J Syst Evol Microbiol in press 10.1099/ijs.0.011148-0 19661515

[B8] De BK Stauffer L Koylass MS Sharp SE Gee JE Helsel LO Steigerwalt AG Vega R Clark TA Daneshvar MI Novel *Brucella *strain (BO1) associated with a prosthetic breast implant infection J Clin Microbiol 2008 46 1 43 49 10.1128/JCM.01494-07 17977982 PMC2224274

[B9] Verger JM Grimont F Grimont PAD Grayon M *Brucella*, a monospecific genus as shown by deoxyribonucleic acid hybridization Int J Syst Evol Microbiol 1985 35 292 295 10.1099/00207713-35-3-292

[B10] Verger JM Grimont F Grimont PA Grayon M Taxonomy of the genus *Brucella* Ann Inst Pasteur Microbiol 1987 138 2 235 238 10.1016/0769-2609(87)90199-2 3606880

[B11] Grimont F Verger JM Cornelis P Limet J Lefevre M Grayon M Regnault B Van Broeck J Grimont PA Molecular typing of *Brucella *with cloned DNA probes Res Microbiol 1992 143 1 55 65 10.1016/0923-2508(92)90034-L 1641513

[B12] Cerri D Ebani VV Pedrini A Nuvoloni R Renzoni G Andreani E Farina R Epididymitis by *Brucella ovis*: experimental infection in virgin ram lambs New Microbiol 1999 22 3 227 231 10423741

[B13] Davis CE Troy SB Brucellosis N Engl J Med 2005 353 10 1071 1072 author reply 1071-1072 10.1056/NEJMc051799 16148300

[B14] Fenkci V Cevrioglu S Yilmazer M Ovarian abscess due to *Brucella melitensis* Scand J Infect Dis 2003 35 10 762 763 10.1080/00365540310015665 14606619

[B15] Pappas G Akritidis N Bosilkovski M Tsianos E Brucellosis N Engl J Med 2005 352 22 2325 2336 10.1056/NEJMra050570 15930423

[B16] Troy SB Rickman LS Davis CE Brucellosis in San Diego: epidemiology and species-related differences in acute clinical presentations Medicine (Baltimore) 2005 84 3 174 187 10.1097/01.md.0000165659.20988.25 15879907

[B17] El-Olemy GM Atta AA Mahmoud WH Hamzah EG Brucellosis in man--II. Isolation of the causative organisms with special reference to blood picture and urine constituents Dev Biol Stand 1984 56 573 578 6489632

[B18] El-Olemy GM Atta AA Mahmoud WH Hamzah EG Brucellosis in man. I. Serological diagnosis Dev Biol Stand 1984 56 565 572 6436114

[B19] Quaife RA Brucellosis in man J Med Lab Technol 1969 26 4 349 357 5355352

[B20] Corbel MJ Recent advances in brucellosis J Med Microbiol 1997 46 2 101 103 10.1099/00222615-46-2-101 9060868

[B21] Al-Anazi AR Aziz S Fouda MA Brucellosis: haemorrhagic pleural effusion Med Princ Pract 2005 14 2 118 120 10.1159/000083924 15785106

[B22] Hatipoglu CA Bilgin G Tulek N Kosar U Pulmonary involvement in brucellosis J Infect 2005 51 2 116 119 10.1016/j.jinf.2004.10.004 16038761

[B23] Ohshimo S Theegarten D Totsch M Moege J Peitgen K Guzman J Costabel U Esophageal sarcoidosis presenting as pseudodiverticulum Sarcoidosis Vasc Diffuse Lung Dis 2008 25 1 64 67 19070263

[B24] Olukman O Pulmonary involvement in childhood brucellosis: a case report Vector Borne Zoonotic Dis 2008 8 2 245 248 10.1089/vbz.2007.0185 18260787

[B25] Theegarten D Albrecht S Totsch M Teschler H Neubauer H Al Dahouk S Brucellosis of the lung: case report and review of the literature Virchows Arch 2008 452 1 97 101 10.1007/s00428-007-0518-0 17952458

[B26] Webb WA Thoroughman JC Solitary pulmonary nodule due to *Brucella suis*. Report of a case Dis Chest 1966 49 2 222 224 10.1378/chest.49.2.222 5907981

[B27] Park KW Kim DM Park CY Kim HL Jang SJ Choi YS Park MY Song HJ Lee SH Fatal systemic infection with multifocal liver and lung nodules caused by *Brucella abortus* Am J Trop Med Hyg 2007 77 6 1120 1123 18165533

[B28] Alton GG Jones LM Pietz DE Laboratory techniques in Brucellosis Monogr Ser World Health Organ 1975 55 1 163 812265

[B29] Institute/NCCLS CLSI ed. Performance standards for antimicrobial susceptibility testing-19th informational supplement-M100-S19 2009 Wayne, PA: CLSI

[B30] Jevitt LA Weigel LM De B Popovic T Patel JB Development of a broth microdilution procedure for antimicrobial susceptibility testing of *Brucella *spp., abstr. C-357. Abstr105th Gen Meet Am soc Microbiol 2005: 2005; Atlanta, GA 2005 American Society for Microbiology, Washington, D.C.

[B31] Gee JE De BK Levett PN Whitney AM Novak RT Popovic T Use of 16S rRNA gene sequencing for rapid confirmatory identification of *Brucella *isolates J Clin Microbiol 2004 42 8 3649 3654 10.1128/JCM.42.8.3649-3654.2004 15297511 PMC497563

[B32] Paquet JY Diaz MA Genevrois S Grayon M Verger JM de Bolle X Lakey JH Letesson JJ Cloeckaert A Molecular, antigenic, and functional analyses of Omp2b porin size variants of *Brucella *spp J Bacteriol 2001 183 16 4839 4847 10.1128/JB.183.16.4839-4847.2001 11466287 PMC99538

[B33] Scholz HC Al Dahouk S Tomaso H Neubauer H Witte A Schloter M Kampfer P Falsen E Pfeffer M Engel M Genetic diversity and phylogenetic relationships of bacteria belonging to the *Ochrobactrum-Brucella *group by *recA *and 16S rRNA gene-based comparative sequence analysis Syst Appl Microbiol 2008 31 1 1 16 10.1016/j.syapm.2007.10.004 18222618

[B34] Batchelor BI Brindle RJ Gilks GF Selkon JB Biochemical mis-identification of *Brucella melitensis *and subsequent laboratory-acquired infections The Journal of hospital infection 1992 22 2 159 162 10.1016/0195-6701(92)90100-Z 1358958

[B35] Elsaghir AA James EA Misidentification of *Brucella melitensis *as *Ochrobactrum anthropi *by API 20NE J Med Microbiol 2003 52 Pt 5 441 442 10.1099/jmm.0.05153-0 12721322

[B36] Cloeckaert A Grayon M Grepinet O An IS711 element downstream of the bp26 gene is a specific marker of *Brucella *spp. isolated from marine mammals Clin Diagn Lab Immunol 2000 7 5 835 839 10973465 10.1128/cdli.7.5.835-839.2000 PMC95966

[B37] Halling SM Tatum FM Bricker BJ Sequence and characterization of an insertion sequence, IS711, from *Brucella ovis* Gene 1993 133 1 123 127 10.1016/0378-1119(93)90236-V 8224885

[B38] Maquart M Zygmunt MS Cloeckaert A Marine mammal *Brucella *isolates with different genomic characteristics display a differential response when infecting human macrophages in culture Microbes and infection/Institut Pasteur 2009 11 3 361 366 19397885 10.1016/j.micinf.2008.12.012

[B39] Gurtler V Mayall BC Genomic approaches to typing, taxonomy and evolution of bacterial isolates Int J Syst Evol Microbiol 2001 51 Pt 1 3 16 11211268 10.1099/00207713-51-1-3

[B40] Thompson CC Thompson FL Vandemeulebroecke K Hoste B Dawyndt P Swings J Use of recA as an alternative phylogenetic marker in the family Vibrionaceae Int J Syst Evol Microbiol 2004 54 Pt 3 919 924 10.1099/ijs.0.02963-0 15143042

[B41] Scholz HC Tomaso H Dahouk SA Witte A Schloter M Kampfer P Falsen E Neubauer H Genotyping of *Ochrobactrum anthropi *by recA-based comparative sequence, PCR-RFLP, and 16S rRNA gene analysis FEMS Microbiol Lett 2006 257 1 7 16 10.1111/j.1574-6968.2006.00153.x 16553826

[B42] Cloeckaert A Grayon M Verger JM Letesson JJ Godfroid F Conservation of seven genes involved in the biosynthesis of the lipopolysaccharide O-side chain in *Brucella *spp Res Microbiol 2000 151 3 209 216 10.1016/S0923-2508(00)00141-8 10865948

[B43] Cloeckaert A Grepinet O Salih-Alj Debbarh H Zygmunt MS Overproduction of the *Brucella melitensis *heat shock protein DnaK in *Escherichia coli *and its localization by use of specific monoclonal antibodies in *B. melitensis *cells and fractions Res Microbiol 1996 147 3 145 157 10.1016/0923-2508(96)80214-2 8761733

[B44] Cloeckaert A Jacques I Grillo MJ Marin CM Grayon M Blasco JM Verger JM Development and evaluation as vaccines in mice of *Brucella melitensis *Rev.1 single and double deletion mutants of the bp26 and omp31 genes coding for antigens of diagnostic significance in ovine brucellosis Vaccine 2004 22 21-22 2827 2835 10.1016/j.vaccine.2004.01.001 15246618

[B45] Cloeckaert A Verger JM Grayon M Grepinet O Restriction site polymorphism of the genes encoding the major 25 kDa and 36 kDa outer-membrane proteins of *Brucella* Microbiology 1995 141 Pt 9 2111 2121 10.1099/13500872-141-9-2111 7496522

[B46] Kumar S Nei M Dudley J Tamura K MEGA: a biologist-centric software for evolutionary analysis of DNA and protein sequences Brief Bioinform 2008 9 4 299 306 10.1093/bib/bbn017 18417537 PMC2562624

[B47] Whatmore AM Perrett LL MacMillan AP Characterisation of the genetic diversity of *Brucella *by multilocus sequencing BMC Microbiol 2007 7 34 10.1186/1471-2180-7-34 17448232 PMC1877810

[B48] Huynh LY Van Ert MN Hadfield T Probert WS Bellaire BH Dobson M Burgess RJ Weyant RS Popovic T Zanecki S Multiple Locus Variable Number Tandem Repeat (VNTR) Analysis (MLVA) of *Brucella *spp. identifies species specific markers and insights into phylogenetic relatiohsips National Institute of Allergy and Infectious Disease, NIH: Frontiers in Research 2008

[B49] Tiller RV De BK Boshra M Huynh LY Van Ert MN Wagner DM Klena J MT S El-Shafie SS Keim P Comparison of two multiple locus variable number tandem repeat (VNTR) analysis (MLVA) methods for molecular strain typing human *Brucella melitensis *isolates from the Middle East Journal of Clinical Microbiology 2009 47 7 2226 2231 10.1128/JCM.02362-08 19439543 PMC2708484

